# Cardiac valve mass revealed by venous and arterial embolism: case report and review of the literature

**DOI:** 10.1093/ehjcr/ytag224

**Published:** 2026-03-25

**Authors:** Emna Derbel, Naziha Turki, Iheb Kechaw, Rania Hammami

**Affiliations:** Department of Cardiology, Military University Hospital of Sfax, University of Sfax, Thyna, 3083 Sfax, Tunisia; Department of Cardiology, Military University Hospital of Sfax, University of Sfax, Thyna, 3083 Sfax, Tunisia; Department of Cardiology, Polyclinique El Manar, 2092 Tunis, Tunisia; Department of Cardiology, Military University Hospital of Sfax, University of Sfax, Thyna, 3083 Sfax, Tunisia

**Keywords:** Case report, Marantic endocarditis, Mitral valve, Stroke, Paraneoplastic

## Abstract

**Background:**

Intracardiac masses are rare, occurring in approximately 0.1%–0.3% of patients undergoing echocardiography. They include thrombi, vegetations, and tumours. Echocardiographic identification is often challenging and can be misleading.

**Case summary:**

We report a 72-year-old man admitted for concomitant deep vein thrombosis and a transient ischaemic stroke. Transthoracic echocardiography showed a small mass appended to the mitral valve. Transoesophageal echocardiography, performed to investigate paradoxical embolism showed a hyperechoic, mobile mitral mass attached to the leaflets closure line, without mitral regurgitation or stenosis. Valvular fibroelastoma, myxoma, and endocarditis were considered in the differential diagnosis; however, inflammatory markers and microbiological tests were unremarkable. Whole-body CT revealed pancreatic cancer, supporting the diagnosis of marantic endocarditis. The patient received curative anticoagulation with apixaban and was referred to an oncologist. The evolution was favourable after 3 months.

**Discussion:**

We will discuss through our case and a literature review of the distinctive clinical and echocardiographic features of valve mass diagnosis.

Learning points

- Intracardiac masses in patients with concomitant arterial and venous embolism
- To demonstrate the pivotal role of multimodality echocardiography in achieving an accurate diagnosis of valvular masses without the need for additional cross-sectional imaging.
- The effective use of apixaban as anticoagulant therapy in marantic endocarditis

## Introduction

Intracardiac masses are an uncommon clinical finding, detected in approximately 0.1%–0.3% of patients undergoing echocardiography.^1^ Among these, thrombi and vegetations are the predominant causes. Cardiac tumours are rare, with secondary lesions more common than primary ones. Primary cardiac tumours occur in less than 0.1% of cases and are predominantly benign, with myxomas representing the most frequent subtype, accounting for 40%–60%.^2^ Marantic endocarditis (ME), also called nonbacterial thrombotic endocarditis (NBTE), is a rare subset of intracardiac masses characterized by sterile, fibrinous vegetations, often associated with malignancy or systemic disorders. The diagnosis is usually considered following a negative infectious work-up. Despite its strong association with thromboembolic events, NBTE is frequently underrecognized.^3^ Echocardiographic diagnosis of intracardiac masses remains challenging, as echogenic features and morphologies often overlap, necessitating careful assessment for accurate diagnosis and management.^4^ This case is particularly original, as it illustrates marantic endocarditis presenting concurrent venous and arterial thromboembolic events and demonstrates the distinctive echocardiographic properties that enabled diagnosis.

## Summary figure

**Figure:**
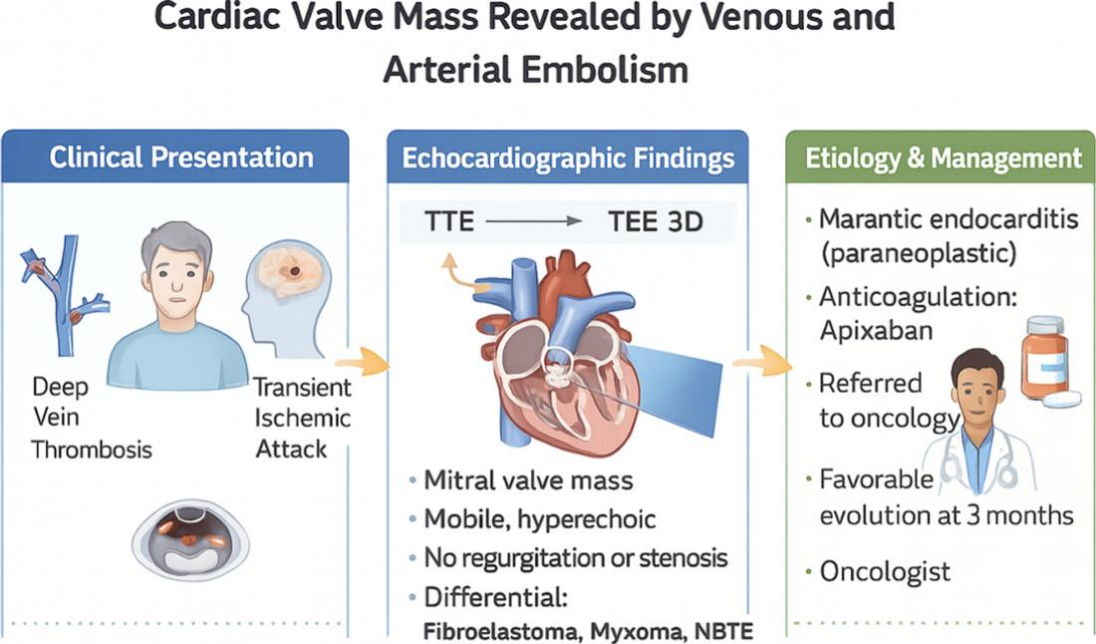


## Case presentation

**Figure 1:**
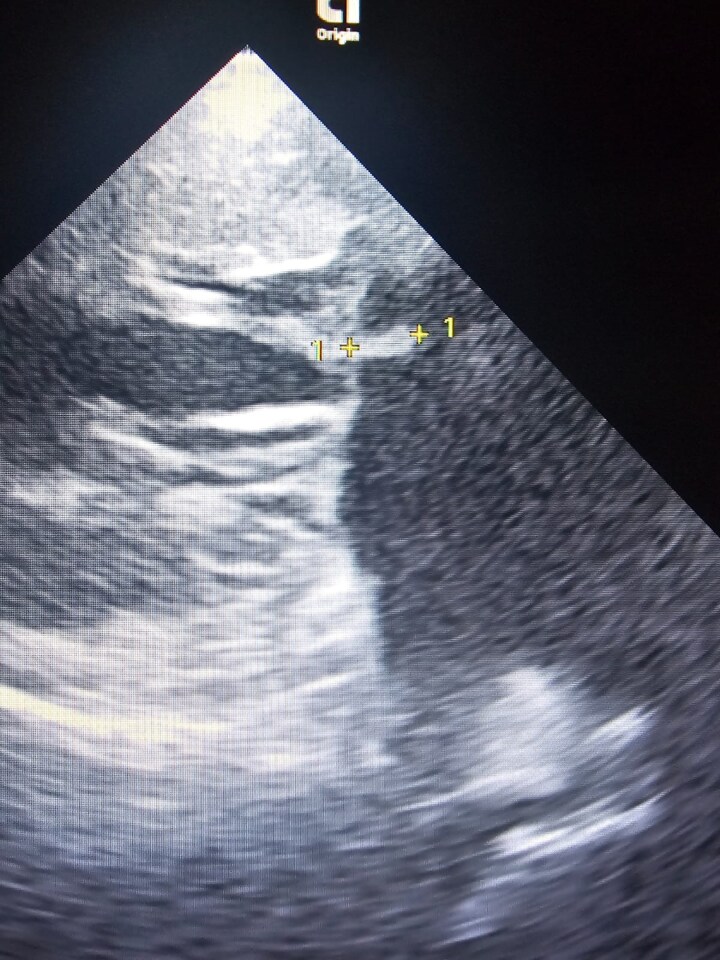
Transthoracic echocardiographic image (parasternal long-axis view): a small echogenic, sessile mass attached to the atrial surface of the anterior mitral leaflet.

**Figure 2:**
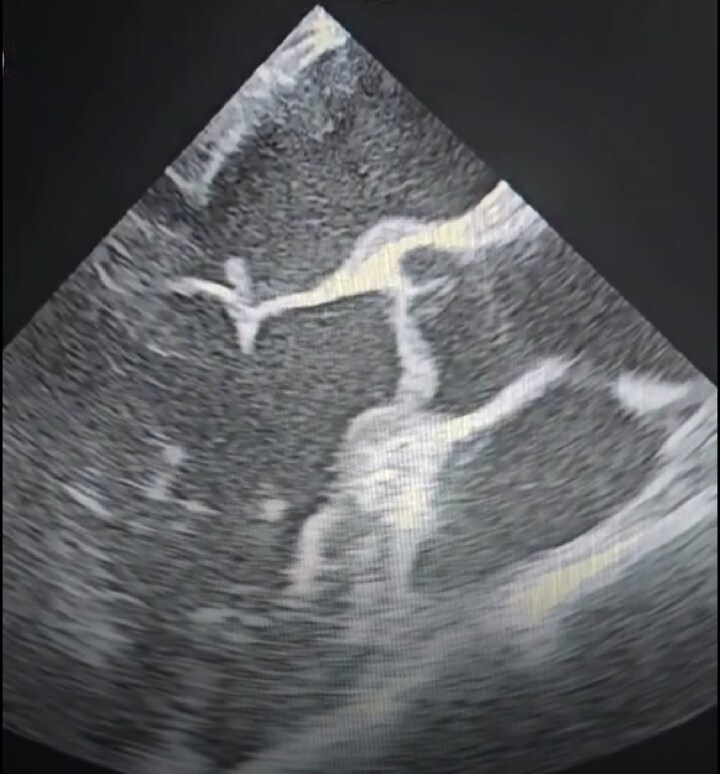
Transoesophageal echocardiographic image: a filamentous, echogenic structure attached to the leaflet coaptation margin in the atrial side of the mitral valve.

**Figure 3:**
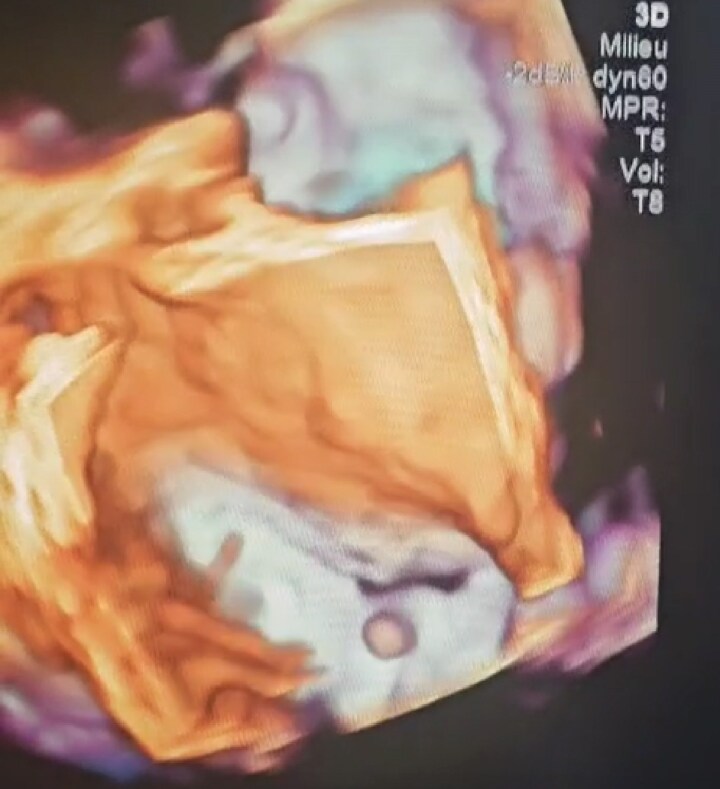
Three-dimensional transoesophageal echocardiographic view: a filamentous mass attached to the atrial surface of the mitral valve. The mitral leaflets are structurally preserved.

A 72-year-old man with type 2 diabetes mellitus and an active smoking history was admitted for management of a distal deep vein thrombosis (DVT) of the left leg. He had no fever or infectious symptoms. Physical examination revealed pain, swelling and erythema of the lower leg. He was haemodynamically stable, with no dyspnoea or respiratory distress. Venous Doppler ultrasound confirmed recent occlusive thrombosis of the left posterior tibial vein. Two days after admission, he developed a transient episode of right-sided hemiparesis without sensory deficit, which resolved completely within minutes, consistent with a transient ischaemic attack (TIA). Brain magnetic resonance imaging (MRI) showed no evidence of ischaemic lesion. Furthermore, there were no peripheral stigmata of infection, and cardiac auscultation revealed no murmurs. As part of the aetiological workup for thromboembolism, 24-h Holter monitoring ruled out supraventricular arrhythmia. In addition, transthoracic echocardiography (TTE) was performed to search for an intracardiac thrombus, mass, or shunt in the context of suspected paradoxical embolism. It revealed a thin, highly echogenic, filamentous, and mobile mass attached to the atrial side of the anterior mitral leaflet, measuring approximately 9 mm in length. No intracavitary thrombus was observed (*Figure 1*). Transoesophageal echocardiography (TEE) provided further characterization, identifying a small, sessile mass adherent to the closure line of the anterior mitral leaflet, with high echogenicity and smooth margins. The lesion demonstrated no valvular thickening, perforation, or abscess formation. Bubble contrast study was negative, excluding a patent foramen oval or intracardiac shunt (*Figure 2*). Three-dimensional echocardiography offered enhanced spatial definition of the mitral valve apparatus, confirming normal leaflet morphology and preserved coaptation, and clearly delineating the attachment site of the mass (*Figure 3*). These echocardiographic features were compatible with either a small valvular fibroelastoma or mitral vegetation. Accordingly, an infectious workup was initiated. Laboratory tests showed normal white blood cell count, mildly elevated C-reactive protein (79 mg/L), and persistently negative procalcitonin. Serial blood cultures remained sterile, and there were no clinical or imaging signs suggestive of infection (Table 1). Given the presence of unexplained weight loss, multiple thromboembolic events, and the absence of infection, a neoplastic aetiology was suspected. A whole-body computed tomography (CT) scan was performed, revealing a heterogeneous hypodense mass in the pancreatic head, suggestive of malignancy and consistent with adenocarcinoma. These findings supported the diagnosis of cancer-associated NBTE. Full dose anticoagulation with apixaban, already initiated for the treatment of DVT, was maintained. He was subsequently referred to oncology for management of pancreatic cancer. During follow-up, serial echocardiography demonstrated complete regression of the mitral mass, and no further embolic events were reported.

**Table 1:** Laboratory data

Biological parameter	Patient result	Reference range
Leukocytes	8.87 × 10^3^/µl	4.0–10.0 × 10^3^/µl
C-reactive protein (CRP)	79 mg/L	<5.0 mg/L
Procalcitonin	0.08 µg/L	<0.1–0.15 µg/L
Blood cultures (three sets)	No growth	—
Urine culture	No growth	—
Fungal blood cultures	No growth	—
Bronchoalveolar lavage culture	Negative	—
Brucella spp. serology	Negative	—
*Bartonella spp. serology*	Negative	—
*Coxiella burnetii* serology	Negative	—
Legionella spp. serology	Negative	—
Mycoplasma spp. Serology	Negative	—

## Discussion

We present a unique case of a valvular cardiac mass, highlighting the clinical approach and echocardiographic findings that can help differentiate NBTE from other potential causes of valve masses, even without the need for additional cardiac imaging.

Cardiac valve masses represent a heterogeneous group of entities, including thrombi, infective and non-infective vegetations, and tumours. They are frequently identified incidentally during routine cardiac imaging, as many patients remain asymptomatic.^1,2^ Despite their low incidence, these lesions pose significant diagnostic and therapeutic challenges because of their potential to cause embolic events or impair cardiac function. Precise differentiation among these entities is crucial, as it directly influences both prognosis and management strategies. Among primary cardiac tumours, benign forms particularly myxomas and papillary fibroelastomas are the most frequently encountered.^4^

ME, is a rare subtype characterized by sterile vegetations on cardiac valves, most frequently the mitral and aortic valves, and less commonly on the right-sided valves.^5,6^ It typically occurs in the context of an underlying hypercoagulable state, most notably advanced malignancy or autoimmune disease.^7^ Among malignancies, adenocarcinomas of the pancreas, lungs and colon are the most frequently associated. In large autopsy series, the prevalence of NBTE has been reported to be between 1.3% and 3.7% of adult populations, reflecting its often subclinical course and the difficulty of establishing an ante-mortem diagnosis.^5,8^

The pathogenesis of NBTE involves a multifactorial mechanism linking systemic inflammation, endothelial injury, and tumour-induced hypercoagulability.^9,10^ This leads to endothelial disruption and subsequent deposition of platelet fibrin aggregates on the valve surface. Diabetes mellitus and active smoking, both present in this case, likely compounded the endothelial dysfunction and hypercoagulability, further predisposing to thrombus formation.

Systemic embolization represents the most frequent clinical manifestation of NBTE, reported in up to 80% of cases.^11^ Emboli often involve the central nervous system, kidneys, spleen, and coronary circulation. Neurological events, particularly ischaemic strokes, are the most common. Our case highlights this original presentation with both DVT and TIA, emphasizing the necessity of considering NBTE in simultaneous thromboembolic phenomena, especially with negative infectious workup and underlying cancer.

Echocardiography remains the cornerstone in detecting intracardiac masses. However, differentiation among thrombi, infectious vegetations, tumours, and NBTE lesions is challenging.^4^ NBTE vegetations are small, sessile, broad-based, mildly mobile with high echogenicity, commonly affecting the upstream side of the valves, without valve destruction or regurgitation. TTE and TEE are generally recommended as first-line imaging modalities for the diagnosis of NBTE.^12^ In addition, three-dimensional TEE can be equally useful, as it offers superior spatial visualization of cardiac structures and valvular lesions, as illustrated in our case. We showed in *Table 2* a comparison of key echocardiographic, clinical, and pathological features of intracardiac masses.

**Table 2:** Key morphological and clinical characteristics of common intracardiac masses

Feature	Papillary fibroelastoma	Myxoma	Infective endocarditis	Marantic endocarditis
Nature of the lesion	Benign cardiac tumour	Benign cardiac tumour	Infectious vegetation	Sterile fibrin-platelet thrombi
Typical appearance	Small pedunculated frond-like mass (‘sea anemone’)	Solitary, gelatinous, heterogeneous mass, often pedunculated	Irregular, heterogeneous, oscillating mass	Small, homogeneous sessile vegetations
Mobility	Highly mobile, attached by a thin stalk	Mobile, may prolapse into chambers	Highly mobile, chaotic motion	Low to moderate mobility
Location	Aortic and mitral valves	Atria (mostly left atrium), can involve valve	Any valve; often mitral > aortic	Mitral and aortic
Attachment site	On downstream side	Typically attached to interatrial septum or atrial wall	Upstream side (flow-dependent)	On leaflet coaptation margin
Size	<1 cm, well defined	Variable, usually 1–6 cm	Variable size	Small (1–10 mm), may be multiple
Valve destruction / perforation	Absent	Absent, rare	Frequent (perforation, annular abscess)	Absent
Associated regurgitation	Rare	May cause obstruction or regurgitation	Common, may be severe	Mild or absent
Risk of embolization	High	High (especially left atrial myxoma)	High	Very high, often recurrent
Clinical context	Often incidental or embolic stroke	Symptomatic (obstruction, embolism)	Fever, positive blood cultures	Cancer, autoimmune disease, hypercoagulable state

Although cardiac CT and MRI can offer complementary tissue characterization, their role in marantic endocarditis is generally limited to cases with inconclusive echocardiographic findings.^13^ In our patient, the diagnosis was accurately established through expert echocardiographic assessment, without the need for additional imaging, underscoring that meticulous evaluation by experienced operators may be sufficient to identify NBTE and guide management.

There is no consensus treatment for NBTE, primarily based on anticoagulation to prevent embolism and treating underlying causes. Traditionally, unfractionated heparin or low molecular weight heparin has been used. Recently, direct oral anticoagulants (DOACs), including apixaban, have gained attention. Studies suggest apixaban's efficacy and safety in cancer-associated thrombosis, with emerging evidence supporting its role in NBTE management.^14,15^ Our case adds clinical experience favouring apixaban use, with mass regression and embolic event resolution. Further randomized studies are warranted to establish DOACs as standard therapy in NBTE. Early oncologic treatment likely contributed to improving the hypercoagulable state and preventing recurrence of valvular thrombi or embolic events.

## Conclusion

This case report illustrates the clinical complexity of intracardiac masses. Confirming diagnosis and differentiation from other types of cardiac masses rely on the integration of a meticulous evaluation of clinical findings and detailed echocardiographic assessment by experienced operators. This approach not only facilitates timely diagnosis but also supports cost-effectiveness. Anticoagulation with apixaban is a promising therapeutic approach in cancer-associated NBTE, but more robust evidence is needed. Multidisciplinary management involving cardiology, oncology, and haematology is critical for optimizing outcomes.

## Data Availability

All relevant data are included in the article. No additional data are available.

## Lead author biography

I am Dr. Emna Derbel, a cardiology specialist working at the Department of Cardiology at the Military University Hospital of Sfax, Tunisia. My clinical expertise includes cardiac imaging, valvular heart diseases, and cardiomyopathies. I am also actively involved in the cardiac catheterization laboratory, with a particular interest in coronary interventional cardiology. I am passionate about research and scientific production, aiming to advance knowledge and improve patient care in both interventional and non-invasive cardiology.

## Notes

**Consent:** The authors confirm that written consent for submission and publication of this case report including images and associated text has been obtained from the patient, in line with COPE guidance.
